# Development of Electronic Nose for Qualitative and Quantitative Monitoring of Volatile Flammable Liquids

**DOI:** 10.3390/s20071817

**Published:** 2020-03-25

**Authors:** Zhiyuan Wu, Hang Wang, Xiping Wang, Hunlong Zheng, Zhiming Chen, Chun Meng

**Affiliations:** 1 College of Biological Science and Engineering, Fuzhou University, Fuzhou 350108, China; wuzhiyuann@163.com (Z.W.); wanghang@fzu.edu.cn (H.W.); wangxipingcn@163.com (X.W.); zhenghunlong@163.com (H.Z.); zhimingchenn@163.com (Z.C.); 2 State Key Laboratory of Photocatalysis on Energy and Environment, Fuzhou University, Fuzhou 350108, China

**Keywords:** electronic nose, flammable liquids, qualitative and quantitative analysis, back propagation artificial neural network

## Abstract

A real-time electric nose (E-nose) with a metal oxide sensor (MOS) array was developed to monitor 5 highly flammable liquids (ethanol, tetrahydrofuran, turpentine, lacquer thinner, and gasoline) in this work. We found that temperature had a significant impact on the test results and temperature control could efficiently improve the performance of our E-nose. The results of our qualitative analysis showed that principal component analysis (PCA) could not efficiently distinguish these samples compared to a back-propagation artificial neural network (BP-ANN) which had a 100% accuracy rate on the test samples. Quantitative analysis was performed by regression analysis and the average errors were 9.1%–18.4%. In addition, through anti-interference training, the E-nose could filter out the potential false alarm caused by mosquito repellent, perfume and hair jelly.

## 1. Introduction

With the acceleration of urbanization worldwide, public transportation systems such as buses are playing an increasingly important role in urban economic development [1]. According to data from the China National Bureau of Statistics, there were 367,292 buses operated in 2008, which increased to 554,820 in 2017 [2]. With the increase in the number of buses, we are facing more serious safety issues. Bus safety accidents caused by arson occurred in both developing and developed countries [3,4]. For example, there are about 70 bus fires per year in Australia, and in Norway, Sweden, and Finland about 1% of all buses in service suffer from a fire or arson incident each year [4]. The most common substances used for bus arson are commercial flammable liquids such as gasoline, ethanol and, lacquer thinner, which are easy to purchase and ignite. According to 41 reports of bus fires in China, more than 58% of the casualties were caused by flammable liquid arson, which caused 91 deaths and 323 injuries [3]. Therefore, monitoring equipment for flammable liquids is extremely important to prevent bus arson.

Detection and analysis of flammable liquids have been developed due to strong demand in many fields, especially in the field of public safety [5,6,7]. The ideal flammable liquid detectors for buses need to have the following characteristics: a recognition model to identify liquid species to prevent false positives, a quantitative model to analyze danger levels, and a temperature controller to manage temperature changes inside the vehicle. The ideal detector also needs superior repeatability, lower-cost, and real-time detection.

Gas chromatography (GC) [8,9] and chromatography/mass spectrometry (MS) [10,11,12,13] are able to accurately recognize and analyze flammable liquids. However, due to drawbacks such as tedious sample pretreatment, long detection time, excessive volumes, and high prices, they are hardly applied on buses [14]. For flammable liquid detection, the metal oxide sensor (MOS) detectors might be applied on buses due to its small size, low cost [15], and real-time detection, such as the HYCWY-01 dangerous gas and liquid detector (produced by Huiwei Security) and the CSD-TWY-CL01 Vehicle Safety Instrument (produced by Zhongshendun Security). In those detectors, the sensor voltage grows as the concentration of flammable liquids grow. The voltage alarm of these two detectors will be triggered when the concentration of the ethanol is higher than 100 ppm. 

However, a single MOS is sensitive to a large number of target materials, such as leather, perfume, etc. Lowering the alarm threshold to increase detection sensitivities may trigger false alarms due to interferences. In contrast, raising the alarm threshold to decrease detection sensitivities may neglect dangerous liquids with lower sensitivities. Fortunately, the use of an E-nose with different cross-reactivity array sensors can improve the specificity of semiconductor sensors for gas recognition and overcome the shortcomings of single-sensor detectors [16,17].

Electronic olfactory systems, known as “E-nose”, can be implemented to analyze various gas components based on a sensing method similar to the human olfactory system [18,19,20,21,22]. In the field of fire safety, Luo et al. developed an E-nose that had successfully classified four industrial gas included CO_2_, CH_4_, NH_3_, VOCs [23]. Sun et al created an E-nose that could successfully identify methanol, ethanol and acetone samples [16]. Despite their advantages, those E-noses still have limitations when they are applied on a bus such as being affected by interference in complex environments. As an interference, the temperature could affect the response values of MOS sensors [24] which can be overcome in two common ways: constant temperature control [23,25] and temperature compensation [26,27,28,29,30]. As far as we know, no electronic nose has been reported for detecting flammable liquids on buses. 

In this research, we developed a real-time electronic nose for qualitative and quantitative analysis of flammable liquids to prevent bus arson. According to The list of prohibited flammable and explosive belongings for bus passengers (Beijing) [31], ethanol, tetrahydrofuran, turpentine, lacquer thinner, and gasoline were chosen as experimental samples and analyzed by the E-nose established in our lab. Our E-nose has a sensor array and an MCU (microcontroller unit) with an optimized recognition model that can perform real-time detection. PID (proportion integration differentiation) temperature control was applied to cope with the temperature change inside the vehicle. Back-propagation artificial neural network (BP-ANN) and principal component analysis (PCA) models were selected to classify flammable liquid species and classify mosquito repellent, perfume, and hair jelly samples as interferences to prevent false positives. The univariate regression analysis model was used for the quantitative analysis of flammable volatiles in the air by our E-nose.

## 2. Materials and Methods

### 2.1. Materials

Ethanol, turpentine, and lacquer thinner were purchased from Sinopharm (Shanghai, China). Tetrahydrofuran was purchased from Enox (Changshu, China) and No.95 gasoline was purchased from the gas station of Minhou County, Fuzhou.

**Figure 1:**
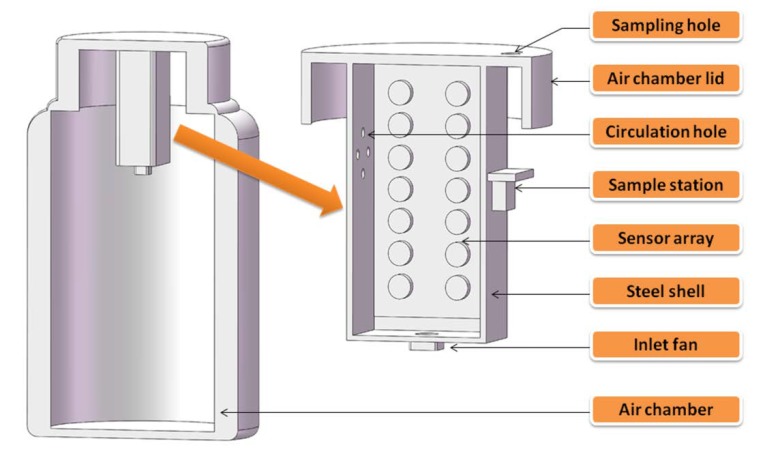
Cross-sectional diagram of the gas chamber and our E-nose.

The E-nose in our laboratory could be divided into six components, including a sampling module, a metal oxide sensor array, a temperature and humidity sensor (DHT22), an MCU (Arduino-mega 2560), a PID-temperature control module, and an operating interface. The sensors named S1-S14 in the array were purchased from FIGARO (Japan) and the relevant information of the sensors is listed in Table 1. These sensors were cross-sensitive to flammable liquids such as ethanol, tetrahydrofuran, and petrol. The sensor array was wrapped in a stainless steel shell (03 L) which was attached to a heating sheet. The heating sheet was controlled by a PID controller and the shell was connected to the lid of the air chamber (10 L). The flow rate of the inlet fan was 52 L/min. Our E-nose and air chamber are shown in Figure 1.

### 2.2. Experimental Methods and Data Process

The experimental process was divided into three parts: sampling, training, and recognition. The initialization time of the E-nose for warming up before detecting/sampling is 5 min.

The sampling part included the following steps. Firstly, the evaporation chamber which was full of fresh air was covered by the lid with the sensor array to detect baseline information. Secondly, a liquid sample was injected into the air chamber through the sampling hole by a pipette. Next, the sampling hole was closed promptly and the flammable liquid began to evaporate quickly. The intake fan would keep drawing the liquid-vapor into the sensor array and then push them to the air chamber through the circulation hole. When the liquid in the gas chamber had completely evaporated, one sampling was complete. Sampling data (sensor voltage, temperature, and humidity) was collected throughout the process above for subsequent analysis. Finally, the lid was opened and the sensors were exposed to fresh air until the voltage of sensors returned to the baseline and the next sampling could be started. After sampling, we could acquire the characteristic values:

ΔV_i_: voltage difference between steady-state and baseline of each metal oxide sensor. (i represent the sensor index).

T: temperature inside the steel shell. 

H: relative humidity inside the steel shell.

An input data could be put into a column vector x as follows:

x = [ΔV_1_ ΔV_2_··ΔV_i_··ΔV_14_ T H]^T^, (1)

All the input data could constitute an input matrix X as follows:

X = [x_1_ x_2_ x_3_···x_n_], (2)

The input matrix of the training set was applied to build recognizing models by MATLAB which were then transplanted to the MCU to achieve real-time detection. During the detection process, the response values of the MOS array and the temperature and humidity detectors were used as inputs and the outputs were the name and concentration of a test sample which were calculated by our recognition model. 

**Figure 2:**
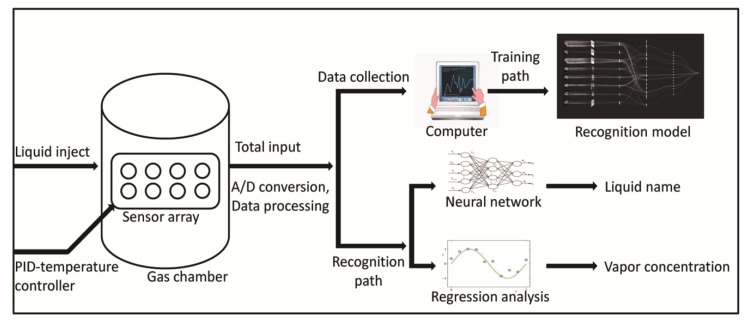
Working principle of the E-nose.

The models of the BP-ANN, PCA, and regressions were built on the matlab2017-a platform. The training process and the recognition process of the E-nose are shown in Figure 2.

In this study, the E-nose was used to collect and analyze five flammable liquid vapors—ethanol, tetrahydrofuran, turpentine, lacquer thinner, and gasoline—with concentrations ranging from 0.70 × 10^2^ ppm to 5.0 × 10^2^ ppm, as well as three interferences that included mosquito repellent, perfumes, and hair jelly with concentrations ranging from 0.90 × 10^2^ ppm to 4.5 × 10^2^ ppm. The temperature range of the working environment was 18–35 °C, the temperature inside the steel shell was controlled by a PID at about 58 ± 3 °C, and the relative humidity inside the steel shell was about 16% ± 10%. The collecting time of each sample was 300–500 s depending on when it completely evaporated. The characteristic values within 100 s of the steady state were taken to represent the corresponding sample and then were collected in the training set. The E-nose collected the voltage value from the sensor array with a sampling frequency of 10 points per second.

### 2.3. Principal Component Analysis (PCA)

In our study, after these five flammable liquids and interfering samples were detected by the E-nose, and PCA as a qualitative method was applied to compute and analyze the 14-dimension characteristic values (ΔV_1_-ΔV_14_) to classify these samples.

### 2.4. Back-Propagation Artificial Neural Network (BP-ANN)

The number of neurons in the input layer was 16, representing 14 metal oxide sensors, temperatures, and humidities inside the shell. The number of neurons in the output layer was 5, representing 5 flammable liquids. The output layer could be expressed by a column vector [A1 A2 A3 A4 A5]^T^ and the output neurons in the training set were set as Table 2. For a training sample, the corresponding element of the output layer was set to 1, and other elements were set to 0. An element error of ± 0.1 was set on the recognition result of the test sample. As a result, elements in test results between 0.9–1.1 could be regarded as positive, and elements between −0.1–0.1 could be regarded as negative. When an element of a test result was positive and others were negative, it could be regarded as the corresponding liquid.

### 2.5. Regression Analysis

In our study, for each kind of flammable liquid, 25 samples (5 concentrations × 5 repetitions) were selected from the training set to construct regression equations by the power law and polynomial law, respectively, between ΔV_i_ and corresponding concentration (ppm). Since the array contained 14 sensors, each kind of liquid will obtain 28 regression equations respectively, including 14 power equations and 14 polynomial equations. Based on the average quantitative errors of the 12 test samples, we could select the most appropriate regression equation to quantify each kind of flammable liquid. After qualitative analysis, the sample concentration can be calculated by the optimal regression equation according to the sample classification.

## 3. Results and Discussion

### 3.1. Odor Data Maps

**Figure 3:**
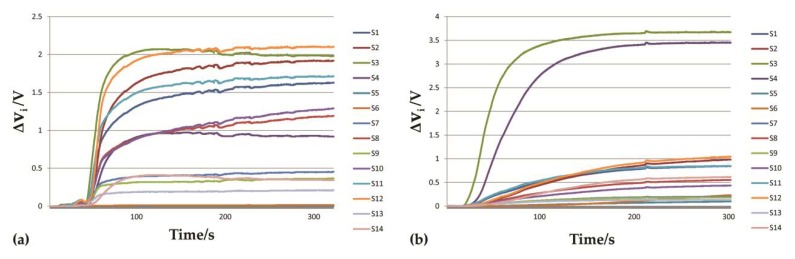
The odor data maps. (**a**) Tetrahydrofuran, (**b**) turpentine. Each curve represents one sensor in the array.

Typical responses of tetrahydrofuran and turpentine called odor data maps (ODM) are shown in Figure 3. The ODM of ethanol, lacquer thinner and gasoline were shown in Figure S1, Figure S2 and Figure S3 (Supplemental Materials 1–3). Firstly, when the sensor was exposed to the fresh air, the baseline voltage was low and stable (the baseline information had been subtracted from Figure 3). After the flammable liquid was injected into the sealed chamber, the voltage response of the sensor increased in line with the liquid’s evaporation. Finally, when the liquid had completely evaporated, the sensor voltage tended to be stable. It can be seen from Figure 3 that the odor data maps of the two samples are different because of their unique physicochemical properties which provide the probabilities of identification of these odors. Furthermore, the response voltages positively relate to the sample concentrations so it is feasible to conduct quantitative regression analysis. 

### 3.2. Temperature Control

**Figure 4:**
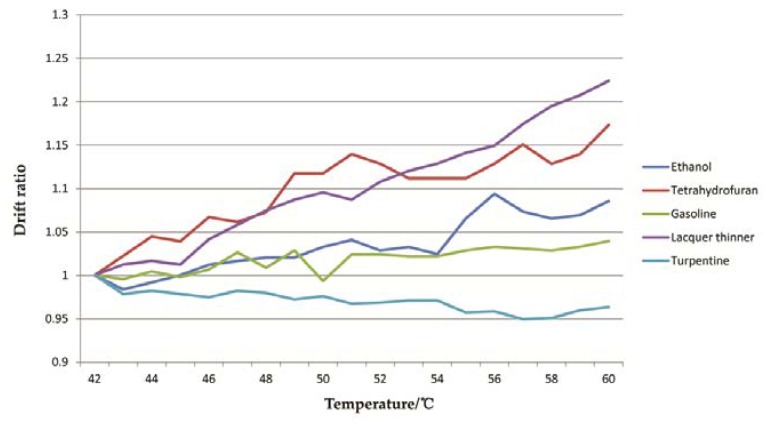
Temperature drift curves of sensor 4.

Because the temperatures inside the vehicle were not constant, measures had to be taken to eliminate the disturbance of temperatures on the response voltage of MOS. One of the measures was temperature compensation which could be compiled by a regression model of the temperatures and the corresponding sensor voltage. However, there was a significant difference among the temperature drift curves of these five liquids as shown in Figure 4, which suggested that the drift pattern relied on the liquid types. Therefore, the temperature compensation is hard to perform when the type of liquid is unknown. 

Another measure was temperature control. A stainless steel shell with a heater controlled by a PID temperature controller was designed to supply constant temperatures for the MOS array. When the indoor temperature ranged from 18 °C to 35 °C, the temperature ranged inside the shell from 40 °C to 58 °C due to the spontaneous heating of MOS. So, the target temperature inside the shell was set to 58 °C and the actual temperature range was about 58 ± 3 °C.

When the room temperatures varied from 18 °C to 35 °C, the classification results of BP-ANN showed that the classification accuracy of the E-nose was only 39% without temperature control and 100% with temperature control. Therefore, temperature control significantly improves the performance of the E-nose and makes it adapt to changing temperatures on buses.

### 3.3. Classification Results of PCA

PCA is an unsupervised linear dimensionality reduction method of multidimensional data analysis that ensures maximum data discrepancies and has no parameter restrictions. It is widely used in machine vision, odor recognition, and other fields. In our study, the total contribution rate of the first three principal components (PCs) was 85.6% which meant three principal components can represent most of the information of the 14-dimensional variables. 

**Figure 5:**
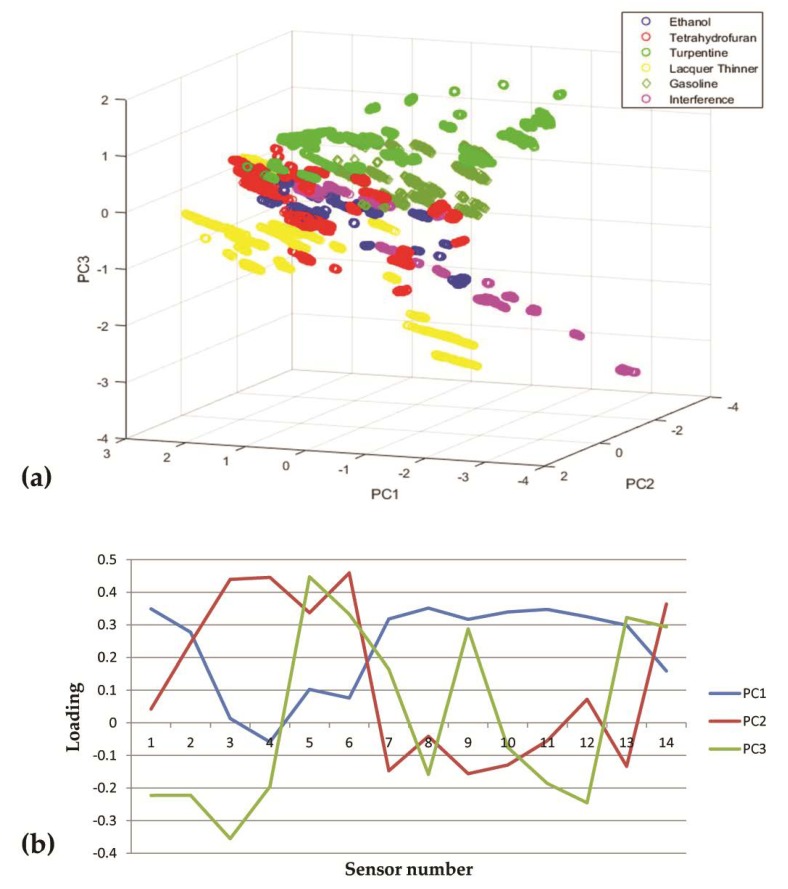
The principal component analysis (PCA). (**a**) Classification results of five flammable liquid samples and three interfering samples, (**b**) loadings of PCA.

As is shown in Figure 5a, the distribution of each kind of sample was not concentrated. There were many cases of sample overlap among them which suggested that these samples were similar so it was difficult to distinguish them without prior knowledge. The influence of each sensor on the three principal components can be seen in Figure 5b. PCA as a traditional unsupervised classification method only restored the basic characteristics of samples. Therefore, it is necessary to resort to a supervised classification method, e.g., BP-ANN, which involves a large number of prior samples for repeated training to improve the classification accuracy.

### 3.4. BP-ANN

#### 3.4.1. Training of BP-ANN

**Figure 6:**
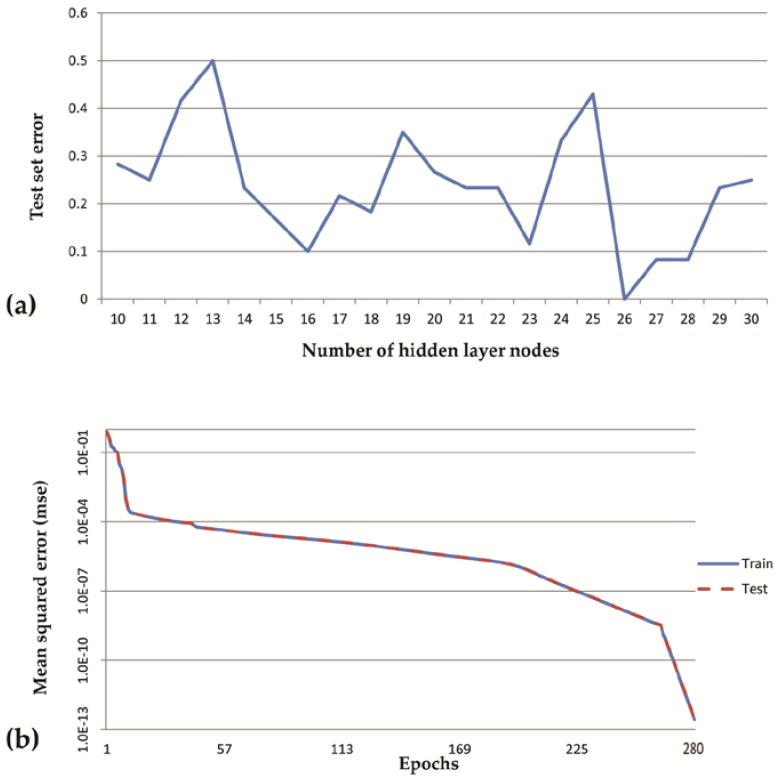
Performance of back propagation artificial neural network. (**a**) Test error in different quantity of hidden nodes, (**b**) training curve of the model with 26 nodes in the hidden layer.

Our BP-ANN model contained three layers: an input layer, a hidden layer, and an output layer. In our study, there were 16 nodes in the input layer and 5 nodes in the output layer. The numbers of hidden layer nodes were variant and could affect network performances. As shown in Figure 6, when the number of hidden layer nodes was 26, the test error was minimal and the network reached the training goal at the 280th iteration and mean-squared error (MSE) reached 2.6383 × 10^−13^. The BP-ANN model designed in this study, with parameters shown in Table 3, achieved a satisfactory simulation precision.

#### 3.4.2. Classification Results of BP-ANN

The BP-ANN as a supervised recognition model continually approached the target by calculating the changes of the network weights and bias in the direction of the slope of the error function. BP-ANN had the learning ability that PCA lacked and had a higher fault tolerance rate than PCA. For each kind of flammable liquid, we selected 12 test samples with their concentrations spread equally in the range of our training set. In total, 60 test samples were predicted by the BP-ANN to test the qualitative accuracy of the electronic nose. In addition, for each kind of interfering liquids, 5 test samples with different concentrations were also tested by the BP-ANN. Moreover, in order to verify the repeatability of the E-nose, 280 ppm ethanol samples were repeatedly tested 5 times and predicted by the BP-ANN. All the test samples were sampled independently of the training samples to verify the performance of the E-nose.

**Figure 7:**
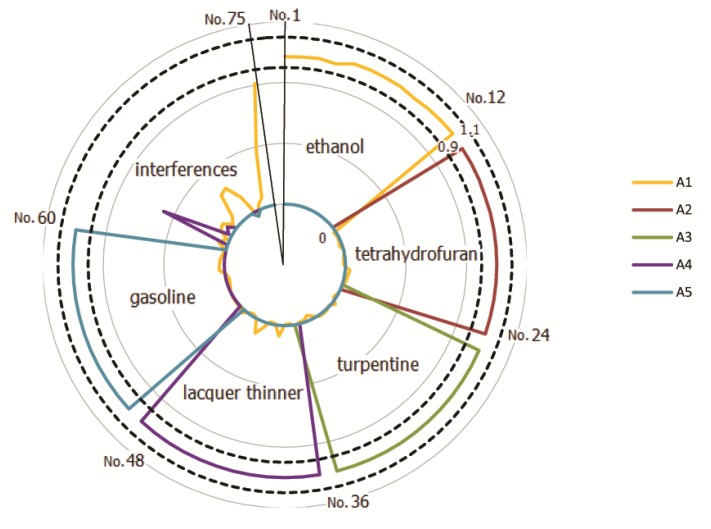
Back propagation artificial neural network (BP-ANN) output layer ([A1 A2 A3 A4 A5]^T^) of 75 test samples. The area between the two dashed lines in the figure is the positive element interval. No.1–No.12 were test samples of ethanol and in each sample, A1 was positive and A2, A3, A4, and A5 were negative. No.13–No.24 were test samples of tetrahydrofuran and in each sample, A2 was positive and A1, A3, A4, and A5 were negative. No.25–No.36 were test samples of turpentine and in each sample, A3 was positive and A1, A2, A4, and A5 were negative. No.37–No.48 were test samples of lacquer thinner and in each sample, A4 was positive and A1, A2, A3, and A5 were negative. No.49–No.60 were test samples of gasoline and in each sample, A5 was positive and A1, A2, A3, and A4 were negative. Furthermore, No.61–No.65 were test samples of mosquito repellent, No.66–No.70 were test samples of perfume and No.71–No.75 were test samples of hair jelly. In each of these 15 interfering samples, A1, A2, A3, A4, and A5 were all negative.

As shown in Figure 7, there was no misjudgment in all these 60 flammable liquid samples. No false-positives were generated among 15 interfering test samples. Five repeated samples were also successfully classified as ethanol. The output values of 75 test samples of BP-ANN were shown in Table S1 (Supplemental Materials 4). Overall, the qualitative accuracy of the test samples was 100%. Therefore, the E-nose has an ability that not only recognizes these five flammable liquids in the concentration range of the training set but also prevents false positives from mosquito repellent, perfumes, and hair jelly within the concentration range of the training set. So, we suggest that it can be applied to complex environments such as buses.

### 3.5. Quantitative Analysis

The ODM of samples showed that the sample concentrations were positively related to sensor voltages, which provided a possibility for the quantitative analysis of the gas concentration. For each kind of flammable liquid, the 12 aforementioned test samples were used to optimize the regression equations according to the average errors of test samples and the results are shown in Table 4.

**Figure 8:**
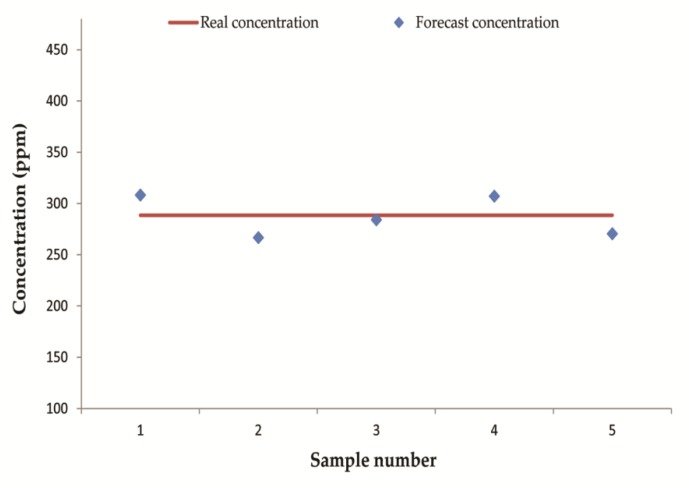
Repeatability test of 5 groups of ethanol.

In each equation in Table 4, the independent variable x represents ΔV_i_ and the dependent variable y represents corresponding concentrations. As shown in Table 4, different types of samples established optimal regression equations with different sensors. The average error of the test set of each liquid was about 12%, indicating that the quantitative ability of our E-nose was relatively accurate. Moreover, in order to verify the repeatability of the E-nose, five repeated experiments of 280 ppm ethanol above-mentioned were predicted by the E-nose. The 5 predict values were located near the real concentration as shown in Figure 8 and the average prediction error of these five experiments is 5.6%. Table 1 shows that the average error of S6 in five repeated experiments of ethanol is 3.6%, which is less than the average prediction error of the 12 groups of ethanol experiments (18.4%). The average error of S6 does not include biases of the regression model, so the quantitative error caused by the biases of the regression equation is higher than the sensor drift. In practical applications, we can design hierarchical alarm thresholds to mitigate the impacts of potential errors in quantitative analysis.

Overall, the E-nose has satisfied quantitative accuracy and is expected to be applied to detect flammable liquids on buses. Based on our results, different alarm threshold values could be set according to actual needs. For example, the hierarchical alarms could be set according to the following concentration levels, 100 ppm: alert of potential prohibited items, 200 ppm: alert of dangerous goods, and 400 ppm: emergency evacuation. However, further studies are required to update our E-nose to identify mixed flammable liquids and mixed system of flammable liquid and interference to expand the application scenario of this E-nose. In addition, the sensitivity of each sensor in the array is different, we can build a segmented regression model based on the different sensors in future work to optimize the regression model and expand the detection range.

## 4. Conclusions

In this paper, 14 metal oxide sensors were used to construct an electronic nose for the qualitative and quantitative detection of five flammable liquids (ethanol, tetrahydrofuran, turpentine, lacquer thinner, and gasoline). Temperature control could greatly improve the performance of our E-nose. The qualitative accuracy rate of the BP-ANN was 100%. The E-nose carrying BP-ANN could not only accurately identify the five flammable liquids but also filter out the potential false alarms of three non-regulated liquids: mosquito repellent, perfume, and hair jelly which was better than PCA. Moreover, the E-nose could detect flammable gas concentrations and the average quantitative error was about 12%. Therefore, combining qualitative and quantitative analysis, electronic noses can monitor flammable liquid threats in buses and provide timely safety warnings. In summary, our E-nose can be used as a new type of bus security equipment. 

## Figures and Tables

**Table 1:** Properties of metal oxide sensors in the array.

Sensor Number	Sensitive Substances	Detection Range (ppm)	Average Error of 5 Repeated Test
S1	H_2_	1–300	1.4%
S2	CO, H_2_S, VOC(volatile organic compounds)	1–500	2.0%
S3	VOC, Tetrahydrofuran, Ethanol	1–10	4.1%
S4	H_2_S, VOC, Turpentine	10–200	9.8%
S5	Stench, H_2_S	500–10,000	9.8%
S6	Ethanol, Stench	1–500	3.6%
S7	Butane, VOC	1–500	15.1%
S8	Petroleum, Gasoline	50–500	2.1%
S9	Methane	50–1000	4.4%
S10	Natural gas, Ethanol, Lacquer thinner	50–1000	2.5%
S11	Tetrahydrofuran	50–7000	1.8%
S12	Ethanol, VOC, Tetrahydrofuran	50–5000	2.5%
S13	Methane, Propane	1–30	5.6%
S14	Butane, Lacquer thinner	1–200	6.0%

**Table 2:** The back-propagation artificial neural network (BP-ANN) output layer of the training set.

Ethanol	Tetrahydrofuran	Turpentine	Lacquer Thinner	Gasoline	Interferences
1	0	0	0	0	0
0	1	0	0	0	0
0	0	1	0	0	0
0	0	0	1	0	0
0	0	0	0	1	0

**Table 3:** Parameters of back-propagation artificial neural network.

Parameter	Value
Training function	Trainbr
Activation function	Sigmoid
Learning rate	0.01
Learning goal (mean-squared error, MSE)	1.0 × 10^−12^
Stopping rule (epoch)	1000

**Table 4:** Univariate regression analysis.

Samples	Sensor (S1-S14)	Regression Equation	*p*-Value (≤0.05)	Average Error
Ethanol	S6	y = 4.788 + 955.6×x − 585.5×x^2^	5.800 × 10^−3^	18.4%
Tetrahydrofuran	S13	y = −134.1 + 183.9×x + 3.961×x^2^	3.252 × 10^−8^	9.7%
Turpentine	S11	y = 106×x^1.884^	2.051 × 10^−7^	9.3%
Lacquer Thinner	S7	y = 75.08 − 62.93×x + 135.6×x^2^	3.216 × 10^−11^	9.1%
Gasoline	S6	y = 5.661 + 927.6×x − 145.8×x^2^	5.076 × 10^−9^	13.7%

## Supplementary Materials

The following are available online at https://www.mdpi.com/1424-8220/20/7/1817/s1. Figure S1: The odor data map (ODM) of ethanol, Figure S2. The odor data map (ODM) of lacquer thinner, Figure S3. The odor data map (ODM) of gasoline, Table S1. back propagation artificial neural network (BP-ANN) output layer of 75 test samples.
