# Supplementary material for: Development of Electronic Nose for Qualitative and Quantitative Monitoring of Volatile Flammable Liquids
Source: Sensors (Basel). 2020 Mar 25;20(7):1817. doi: 10.3390/s20071817 (PMC7180552; doi:10.3390/s20071817)
Supplement: Supplementary file 1 [file sensors-20-01817-s001.pdf]

Supplementary Materials (including three figures and one table)

Development of Electronic Nose for Qualitative and Quantitative Monitoring of Volatile Flammable Liquids

Supplementary Materials 1： The odor data map(ODM) of ethanol

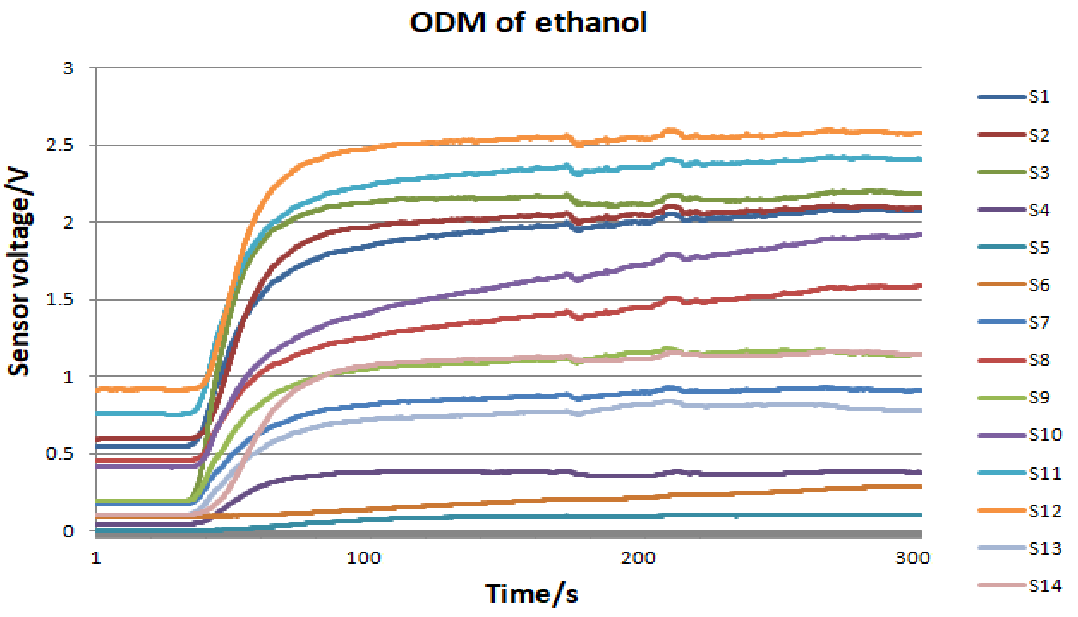

Figure S1. The odor data map(ODM) of ethanol.

Supplementary Materials 2： The odor data map(ODM) of lacquer thinner

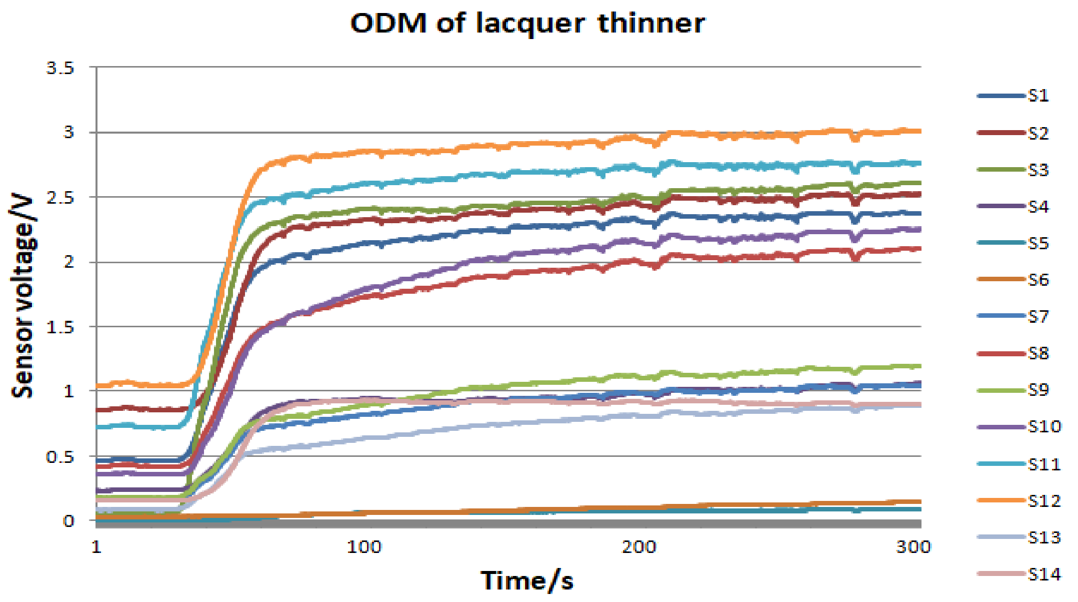

Figure S2. The odor data map(ODM) of lacquer thinner.

Supplementary Materials 3: The odor data map(ODM) of gasoline

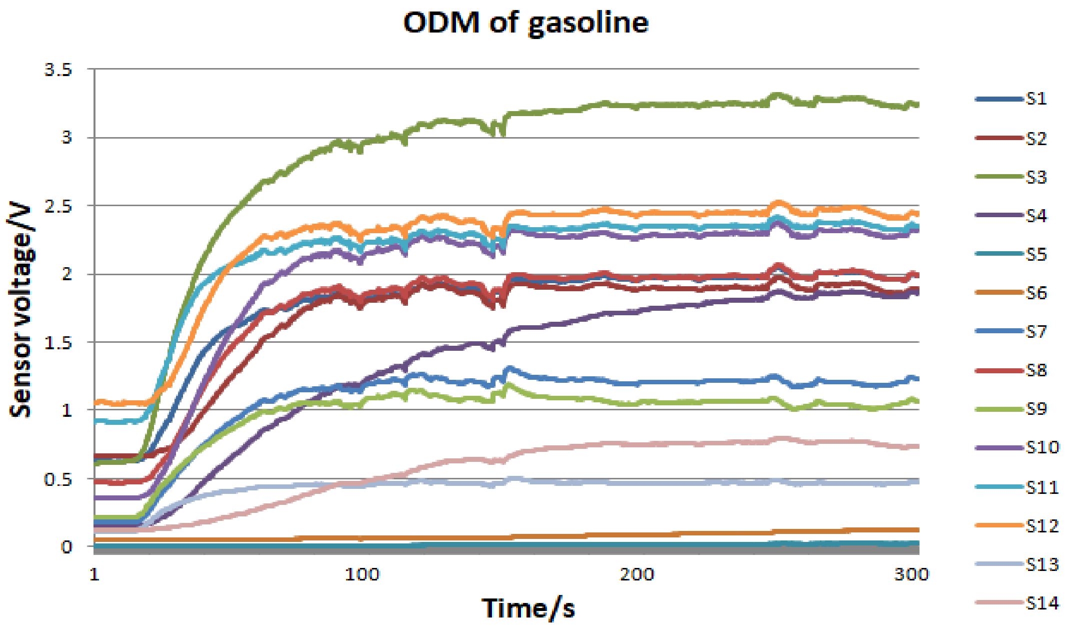

Figure S3. The odor data map(ODM) of gasoline.

Supplementary Materials 4: Table of BP-ANN output layer([A1 A2 A3 A4 A5]^T) of 75 test samples(with different concentration).

There are 75 rows in Table 1 and each row represents a test sample. Element A1–A5 represent ethanol, tetrahydrofuran, turpentine, lacquer thinner, and gasoline respectively. Elements in each row between 0.9–1.1 can be regarded as positive, and elements between -0.1–0.1 can be regarded as negative. When an element in a row is positive and others are negative, it can be regarded as the corresponding liquid.

No.1–No.12 were test samples of ethanol and in each row, A1 was positive and A2, A3, A4, and A5 were negative. No.13–No.24 were test samples of tetrahydrofuran and in each row, A2 was positive and A1, A3, A4, and A5 were negative. No.25–No.36 were test samples of turpentine and in each row, A3 was positive and A1, A2, A4, and A5 were negative. No.37–No.48 were test samples of lacquer thinner and in each row, A4 was positive and A1, A2, A3, and A5 were negative. No.49–No.60 were test samples of gasoline and in each row, A5 is positive and A1, A2, A3, and A4 were negative. Furthermore, No.61–No.65 were test samples of mosquito repellent, No.66 - No.70 were test samples of perfume and No.71–No.75 were test samples of hair jelly. In each row of these 15 interference samples, A1, A2, A3, A4, and A5 were all negative. So, there is no misjudgment in all these 60 flammable liquid samples and no false-positives were generated among 15 interference test samples.

Table S1. BP-ANN output layer([A1 A2 A3 A4 A5]^T) of 75 test samples.

	A1	A2	A3	A4	A5
No.1	0.970252	-1.25E-05	-1.01E-06	5.25E-06	-1.49E-05
No.2	0.972865	-7.83E-06	-6.34E-07	1.03E-05	-8.90E-06
No.3	0.976666	-7.76E-06	-8.61E-07	9.97E-06	-8.16E-06
No.4	0.964942	-1.05E-05	-8.89E-07	1.32E-05	-9.36E-06
No.5	0.999771	3.02E-07	-2.50E-06	6.16E-07	-2.16E-06
No.6	1.002291	2.44E-06	1.28E-06	-1.49E-06	-1.54E-06
No.7	1.000176	8.44E-07	-4.74E-06	1.78E-06	1.65E-06
No.8	1.000614	-1.75E-06	-4.20E-06	2.72E-06	-9.88E-07
No.9	0.989673	9.29E-07	9.23E-07	1.81E-05	-1.12E-07
No.10	0.997234	8.77E-06	2.75E-06	2.32E-05	-2.26E-07
No.11	1.001716	1.34E-05	1.72E-06	7.23E-05	1.20E-06
No.12	1.006325	1.65E-05	9.12E-07	1.35E-04	2.03E-06
No.13	-0.01195	1.00E+00	-7.83E-07	-5.15E-07	-3.12E-06
No.14	-0.00761	0.99997	-1.10E-06	-1.31E-06	-2.75E-06
No.15	-0.01003	0.999861	5.46E-07	-8.18E-08	-2.33E-06
No.16	-0.01851	0.996475	3.97E-06	1.12E-06	-2.61E-06
No.17	0.002428	1.000005	1.39E-06	-3.84E-06	-2.81E-06
No.18	-0.00606	1.000001	-3.53E-07	-2.41E-06	-3.62E-06
No.19	-0.01148	1.000001	1.52E-06	-4.07E-07	-3.08E-06
No.20	-0.01108	0.999952	2.06E-06	-2.96E-07	-3.12E-06
No.21	0.030408	0.999993	3.19E-05	-6.90E-07	-5.14E-06
No.22	0.021728	1.000004	-6.68E-07	-1.70E-06	-2.88E-06
No.23	0.019407	1.000002	-3.00E-06	-2.70E-06	-1.57E-06
No.24	0.007455	1	-3.76E-06	-2.38E-06	-5.54E-06
No.25	0.003932	1.00E-05	1.00E+00	-5.66E-06	-2.01E-05
No.26	-0.00341	-3.34E-06	1.000008	2.67E-07	-6.67E-06
No.27	-0.0156	-3.83E-06	1.000007	-1.57E-06	-1.42E-06
No.28	0.022121	-2.94E-06	1.000008	-4.92E-06	-3.10E-07
No.29	-0.00593	1.40E-05	1.000013	-5.27E-06	-2.61E-05
No.30	0.008843	3.76E-06	1.000003	-2.71E-07	-7.07E-06
No.31	0.009709	-3.88E-06	1.000005	2.17E-06	8.70E-07
No.32	0.007534	-5.66E-06	1.000007	2.61E-06	3.18E-07
No.33	-0.01719	4.53E-06	1.000013	1.20E-06	1.77E-04
No.34	-0.03752	-1.83E-08	1.00001	-2.28E-06	-1.46E-05
No.35	-0.01478	-5.08E-06	1.000007	3.20E-06	-3.78E-06
No.36	-0.01153	-4.76E-06	1.000008	2.07E-06	-2.13E-06
No.37	0.008853	-3.86E-06	-2.98E-06	1.00E+00	-1.12E-05
No.38	-0.01263	-2.88E-06	3.39E-06	1.000009	-6.52E-06
No.39	-0.00593	-1.51E-05	6.44E-06	1.000022	-6.53E-06
No.40	0.069838	-1.72E-05	4.65E-06	1.000025	-5.98E-06

	A1	A2	A3	A4	A5
No.41	-0.00943	-5.33E-07	-2.07E-06	0.999997	-6.32E-06
No.42	-0.01268	-8.29E-06	4.74E-06	1.000013	-5.67E-06
No.43	0.036717	-1.38E-05	5.31E-06	1.00002	-5.78E-06
No.44	0.089705	-1.12E-05	3.18E-06	1.000019	-4.21E-06
No.45	-0.00388	-5.80E-06	-7.60E-07	1.000009	-1.07E-05
No.46	-0.01711	-4.50E-06	4.42E-06	1.000015	-7.18E-06
No.47	-0.00325	-1.38E-05	6.55E-06	1.000025	-6.75E-06
No.48	0.029687	-1.68E-05	6.99E-06	1.000029	-6.29E-06
No.49	0.007632	-2.17E-06	2.45E-06	-3.35E-06	1.00E+00
No.50	0.004735	7.99E-06	9.97E-06	-8.38E-06	1.000012
No.51	0.006226	9.74E-06	1.18E-05	-6.33E-06	1.000014
No.52	0.002486	8.60E-06	1.25E-05	-8.64E-07	1.000014
No.53	-0.00744	1.15E-04	5.88E-04	4.02E-06	1.000012
No.54	-0.01801	-1.47E-05	5.19E-05	7.56E-06	1.000016
No.55	-0.02193	-1.47E-05	8.36E-05	7.77E-06	1.000014
No.56	-0.00956	-1.04E-07	6.00E-05	-5.57E-06	0.999896
No.57	0.035334	-2.59E-06	2.26E-05	-8.17E-06	0.999988
No.58	0.03208	1.72E-06	5.34E-05	-2.52E-06	0.999979
No.59	0.027573	-5.88E-07	6.32E-05	-1.76E-06	0.999993
No.60	0.017601	3.17E-06	9.67E-05	1.93E-06	0.999972
No.61	0.009052	3.04E-06	-3.43E-06	-1.64E-05	-1.38E-06
No.62	0.06206	6.73E-06	-3.06E-06	0.004649	-1.47E-06
No.63	0.020333	5.88E-06	-1.63E-07	0.474541	-1.08E-05
No.64	0.081595	8.83E-06	-2.31E-08	0.022787	-4.62E-06
No.65	0.09082	9.05E-06	1.27E-06	0.054357	-6.20E-06
No.66	0.035034	4.15E-05	7.22E-06	-8.05E-06	1.67E-06
No.67	0.071798	-8.20E-06	9.56E-07	-8.41E-07	3.13E-06
No.68	0.221501	-2.08E-05	6.83E-06	2.14E-05	1.85E-06
No.69	0.2388	-2.53E-05	6.37E-06	2.62E-05	6.90E-07
No.70	0.132236	-8.18E-06	2.38E-06	8.01E-06	2.24E-06
No.71	-0.03855	-8.58E-06	2.94E-05	1.15E-05	-0.03603
No.72	0.033047	-2.17E-05	1.43E-05	2.54E-05	-2.02E-05
No.73	0.071195	-2.40E-05	1.39E-05	2.83E-05	-1.36E-05
No.74	0.37228	-2.29E-05	9.43E-06	2.95E-05	-5.59E-06
No.75	0.81066	-6.34E-06	3.00E-06	1.25E-05	5.02E-06
